# Cerebral MRI Mimicking Pachymeningeal Involvement Associated with Intrathecal Treatment

**DOI:** 10.4274/tjh.galenos.2020.2020.0376

**Published:** 2020-11-19

**Authors:** Semra Paydaş, Kenan Bıçakçı

**Affiliations:** 1 Çukurova University Faculty of Medicine, Department of Oncology, Adana, Turkey

**Keywords:** Meningeal leukemia, MR imaging, leptomeningeal involvement

## To the Editor,

**Figure 1:**
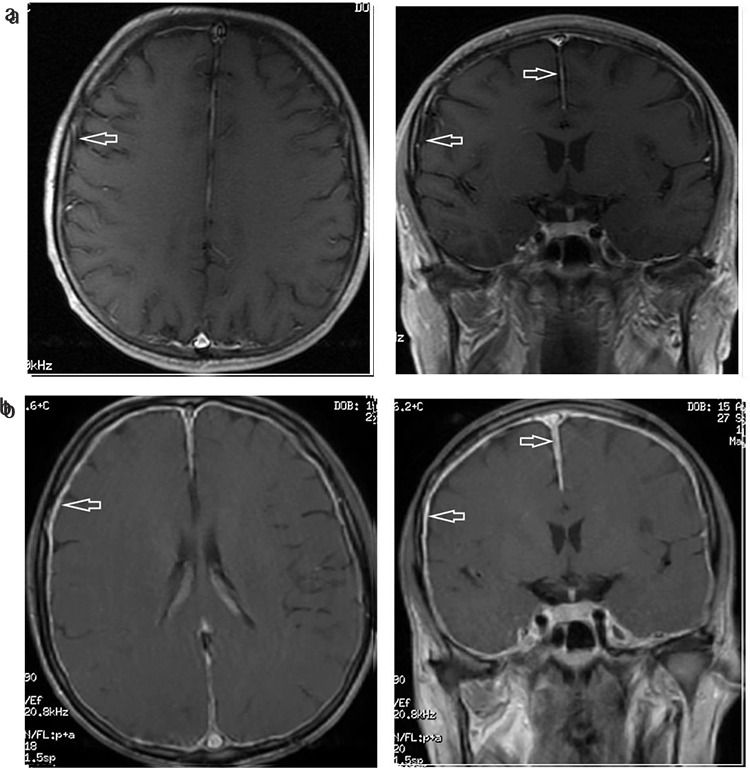
Cerebral MRI revealed leptomeningeal involvement (a), but there was no evidence of meningeal involvement upon cerebral MRI after 2 months (b).

A 43-year-old man was diagnosed with Burkitt’s lymphoma. He had no evidence of central nervous system (CNS) involvement. He was treated with a CODOX-M/IVAC regimen with excellent response. In the last cycle, he complained of nausea and hiccups the day after intrathecal treatment. Cerebral magnetic resonance imaging (MRI) revealed leptomeningeal involvement (Figure 1a). There was no evidence of lymphomatous infiltration in the cerebrospinal fluid sample cytologically, flow cytometrically, or biochemically. His symptoms resolved within 3 days and he was accepted as having a leptomeningeal reaction associated with intrathecal treatment. There was no evidence of meningeal involvement upon cerebral MRI after 2 months (Figure 1b).

Cerebral MRI is the most commonly used imaging method in cases of CNS involvement [1,2]. Leptomeningeal involvement is a relatively rare but important clinical entity and it necessitates intrathecal treatment [3]. However, intrathecal treatment may be used in cases without leptomeningeal involvement for the aim of prophylaxis, such as in cases of systemic lymphoma with high risk of CNS involvement like our patient with Burkitt’s lymphoma [4]. We used intrathecal treatment in the first 3 cycles without complications. However, in the last cycle, the patient complained of vertigo and we ordered MRI with the suspicion of lymphoma. There was no evidence of cerebral lymphoma, but the radiologists reported leptomeningeal involvement. It is well known that contrast-enhanced MRI of the brain showing patchy meningeal enhancement and thickening is suggestive of pachymeningitis and also chronic infections [5,6].

Our case suggests that intrathecal treatment may mimic meningeal involvement and this must be remembered for patients receiving intrathecal drugs.
